# AI-based cluster analysis enables outcomes prediction among patients with increased LVM

**DOI:** 10.3389/fcvm.2024.1357305

**Published:** 2024-09-02

**Authors:** Ranel Loutati, Yotam Kolben, David Luria, Offer Amir, Yitschak Biton

**Affiliations:** Heart Institute, Hadassah Medical Center and The Faculty of Medicine, Hebrew University of Jerusalem, Jerusalem, Israel

**Keywords:** artificial intelligence, cluster analysis, left venticular hypertrophy, cardiovascular outcome assessment, unsupervised learning

## Abstract

**Background:**

The traditional classification of left ventricular hypertrophy (LVH), which relies on left ventricular geometry, fails to correlate with outcomes among patients with increased LV mass (LVM).

**Objectives:**

To identify unique clinical phenotypes of increased LVM patients using unsupervised cluster analysis, and to explore their association with clinical outcomes.

**Methods:**

Among the UK Biobank participants, increased LVM was defined as LVM index ≥72 g/m^2^ for men, and LVM index ≥55 g/m^2^ for women. Baseline demographic, clinical, and laboratory data were collected from the database. Using Ward's minimum variance method, patients were clustered based on 27 variables. The primary outcome was a composite of all-cause mortality with heart failure (HF) admissions, ventricular arrhythmia, and atrial fibrillation (AF). Cox proportional hazard model and Kaplan-Meier survival analysis were applied.

**Results:**

Increased LVM was found in 4,255 individuals, with an average age of 64 ± 7 years. Of these patients, 2,447 (58%) were women. Through cluster analysis, four distinct subgroups were identified. Over a median follow-up period of 5 years (IQR: 4-6), 100 patients (2%) died, 118 (2.8%) were admissioned due to HF, 29 (0.7%) were admissioned due to VA, and 208 (5%) were admissioned due to AF. Univariate Cox analysis demonstrated significantly elevated risks of major events for patients in the 2nd (HR = 1.6; 95% CI 1.2–2.16; *p* < .001), 3rd (HR = 2.04; 95% CI 1.49–2.78; *p* < .001), and 4th (HR = 2.64; 95% CI 1.92–3.62; *p* < .001) clusters compared to the 1st cluster. Further exploration of each cluster revealed unique clinical phenotypes: Cluster 2 comprised mostly overweight women with a high prevalence of chronic lung disease; Cluster 3 consisted mostly of men with a heightened burden of comorbidities; and Cluster 4, mostly men, exhibited the most abnormal cardiac measures.

**Conclusions:**

Unsupervised cluster analysis identified four outcomes-correlated clusters among patients with increased LVM. This phenotypic classification holds promise in offering valuable insights regarding clinical course and outcomes of patients with increased LVM.

## Introduction

Increased left ventricular mass (LVM), often termed in its more severe manifestations as left ventricular hypertrophy (LVH), represents a maladaptive response to various cardiovascular insults, arising from both cardiac and systemic etiologies (1, 2). This morphological alteration is a well-established independent predictor of major cardiovascular events (3–6) and mortality (7, 8). Furthermore, it was intriguingly recognised as a potential treatable target with prognostic implications in clinical trials for hypertension treatment (9). Cardiac magnetic resonance imaging (CMR) is an accurate and reproducible tool for the assessment of LVM, offering additional data regrading the shape, size, and possible pathologies of the left ventricle (10, 11).

The conventional four-tiered classification of LVH patients was initially purposed by Khouri et al., based on the CMR images of patients from the Dallas Heart Study (DHS) (12). This classification is founded on the geometry of the left ventricle, and exhibits a strong link to the pathophysiological mechanisms of pressure and volume overload, which act as the driving forces behind LVH development (13). However, this classification has not consistently shown correlations with clinical outcomes in population-based studies (14, 15), possibly due to its limited consideration of accompyning co-morbidities and clinical features. Hence, there is a need for the identification of high-risk patients among the group of individuals with increased LVM.

Unsupervised cluster analysis plays a pivotal role in the identification of distinct disease phenotypes, proving particularly advantageous when exploring cohorts characterized by heterogeneity (16–18). Therfore, the objectives of this study were to utilize cluster analysis to identify various disease phenotypes among patients with increased LVM within the heterogenous, low-risk population of the UK Biobank participants. Additionally, we aimed to investigate potential correlations between the identified phenotypes and significant clinical outcomes. We sought to explain our findings through cluster exploration and shed light on the characteristics of each cluster using explainability methods.

## Methods

### Study population

Our analysis was performed on a dataset consists of information obtained from the UK Biobank, which is a population-based prospective cohort comprising more than half a million participants aged 37–73, who were recruited in the United Kingdom between 2006 and 2010 (19). During recruitment, extensive data were collected through questionnaires, physical measurements, and biological samples. Furthermore, ongoing data collection was conducted in subsets of the cohort, including repeated assessments and multimodal imaging. To monitor health outcomes, all participants are followed up through linkage to national health-related databases (updated through May 25, 2023). All UK Biobank participants provided written informed consent, and the analysis of the UK Biobank data was approved by the Hadassah Medical Center institutional review board (application 96272).

### Cardiac magnetic resonance acquisition

For this study, we incorporated participants who had undergone cardiac magnetic resonance (CMR) imaging as part of the UK Biobank imaging assessment, and for whom the comprehensive CMR dataset was accessible for retrieval as of January 1, 2023. A comprehensive description of the entire CMR protocol employed by the UK Biobank has been previously documented in meticulous detail (20). In summary, all CMR examinations were conducted within the United Kingdom using a clinical wide-bore 1.5 Tesla scanner (MAGNETOM Aera, Syngo Platform VD13A; Siemens Healthineers, Erlangen, Germany). The acquisition techniques employed a balanced steady-state free precession with typical parameter settings.

### LVM estimation and increased LVM definition

The estimation of LVM using myocardium contours from the InlineVF algorithm was previously described (21). Indexing for body surface area was performed using the DuBois formula (22). The definition of increased LVM is based on previously established cutoffs within the UK Biobank (23): LVM index ≥72 g/m^2^ for men, and LVM index ≥55 g/m^2^ for women. These values represent measurements outside the 95% prediction interval for all age groups.

### Co-morbidities definitions

Co-morbidities were defined using self-report on diseases and drugs, and inpatient ICD-9/10 codes. Each diagnosis was timestamped in order to distinguish between clinical data that was known prior to the CMR and subsequent increased LVM diagnosis, which was used for clustering purposes.

### Outcomes

The primary outcome comprised a composite of all-cause mortality along with admissions due to heart failure (HF), ventricular arrhythmia (VA), and atrial fibrillation (AF). Secondary outcomes included individual occurrences of all-cause mortality, HF admissions, VA, and AF. These outcomes were defined using self-reported data on diseases and drugs, as well as inpatient ICD-9/10 codes. The baseline for outcome definition was established at the time of the participant's CMR study at the UK Biobank assessment center. Participants were followed for the incidence of HF/VA/AF events, death, or until the end of the follow-up period (November 27, 2022), whichever came first.

### Unsupervised clustering

In order to decide how many clusters we aim to find, we utilized a few commonly used methods for deciding the cutoff that dictates the number of clusters. First we use the singular value decomposition (SVD) decomposition to observe the singular values of the data matrix. Essentialy, the singular values represent the data matrix core and allow us to easily perform calculations of needed indices that assist in choosing the number of clusters. Based on these values 2 methods can be used. The former is the Elbow-method, in which the singular values are plotted as bars, and an “elbow” is visually identified as they descent. The point where the reduction in distortion, that represent and error calculation, starts to slow down creates an “elbow” shape on the plot, and it's often where the optimal number of clusters lies. The latter is to observe how many components of the singular values are needed in order to explain at least 90% of the explained variance. In the domain of tabular data, the common rule of thumb is trying to explain 90%, as opposed to other applications like in visual or auditory data, where higher percentages are pursued. In order to add more certainty to our choosing, three commonly used algorithms that inspect the wellness of the clustering were used. The first was Silhouette Coefficient, that measures the quality of clustering by considering both the compactness of clusters and the separation between clusters. It ranges from −1 to 1, where higher values indicate better clustering. A value close to 1 suggests that the clusters are well-separated and compact, indicating a good clustering solution. The second method was Calinski-Harabasz Index. The index, also known as the variance ratio criterion, quantifies the ratio of between-cluster dispersion to within-cluster dispersion. Higher values of the index indicate better clustering quality. A higher value suggests that the clusters are more compact and well-separated from each other. The third algorithm was Davies-Bouldin Index, which evaluates both the compactness of clusters and the separation between clusters. Lower values of the index indicate better clustering quality. A lower value suggests that the clusters are more compact and well-separated, indicating good clustering. For the unsupervised clustering analysis, we employed a widely used algorithm, agglomerative clustering. Agglomerative clustering is a hierarchical method that begins with individual data points and gradually merges them into clusters based on their similarity. Initially, each data point is treated as a separate cluster, and then, using a chosen distance metric, clusters are iteratively merged based on their proximity until a desired number of clusters or a specified stopping criterion is reached. Specifically, we applied Ward's minimum variance method that aims to minimize the variance within each cluster as new data points are added. The result of the hierarchical structuring is presented via a dendrogram, and is then colored in a fixed number of colors, according to the number of clusters chosen by the methods aforementioned above. After specifying the number of clusters and creating them, we proceed with creating a heat map of all the features in the data, calculated per cluster. Since some of the columns in the data are the results of bloodwork, their values were converted to binary values, where 1 indicating an abnormal value, and 0 is in the normal range. Continuous and discrete columns in the data then were processed differently. For the continuous features we calculated the aggregated means per cluster. In order to provide the same scale as the binary data, we reported for each mean its respective quantile from all the observations, providing a value between 0 and 1. For the binary columns, we calculated for each cluster the ratio of number of abnormalities in the cluster over all abnormalities, per feature. Thus we guarantee these numbers sum up to 1, which means that we can distinguish if any cluster has a more prominent percentages of abnormal diagnoses compared to the others. The values for both types of data were then presented in a 2-dimensional table, where the x-axis represents the clusters ($C_{1},\;\ldots C_{n_{clusters}}$), and the y-axis represents the features ($F_{1},\;F_{2},\;\ldots$). The range 0–1 was divided to 5 even segments and a respective color, and each cell was colored according to its respective segment color, in order to visually express the qualities that identify each cluster uniquely.

### Statistical analysis

Continuous variables were expressed as mean ± standard deviation if normally distributed or median with interquartile range if skewed. Categorical variables were presented as frequency (%). Differences between the four clusters were analyzed using one-way ANOVA for continuous variables that were normally distributed, while the Kruskal–Wallis test was used to compare continuous variables that did not adhere to a normal distribution. Multiple comparisons for continuous and categorical variables were tested using Bonferroni's correction. For survival analysis patients were censored only in the case of death. The probability of outcome according to the study groups was graphically displayed according to the method of Kaplan–Meier, with a comparison of cumulative event across strata by the log-rank test. Univariate and multivariate Cox proportional hazards regression modeling were used to compare patients in each of the four clusters, with adjustment made to the strongest predictor of mortality in LVH patients which is LVM (as a continuous variable). Evaluations of the associations between certain features and the composite outcome was also performed by using univariate Cox modeling. All needed assumptions for the use of Cox modeling were checked before a model was fitted. Unsupervised cluster analyses was performed using Python software version 3.11. All statistical analyses were performed using R software version 3.4.4 (R Foundation for Statistical Computing). An association was considered statistically significant for a two-sided *P* value of less than 0.05.

## Results

### Study population and baseline characteristics

Among the 60,650 UK-Biobank participants who underwent CMR, 4,255 individuals met the cutoff for increased LVM diagnosis. The final study had an average age of 64 ± 7 years, of whom 58% were women. The average body mass index (BMI) was 28 (5) which indicated that as an average, the study population was over-weight. The most common co-morbidity was hypertension, that was present in 47% of patients. Other common co-morbidities included dyslipidemia (30%), peripheral vascular disease (26%), and diabetes mellitus (8%). Cardiac measures that were calculated based on the CMR images included left ventricular ejection fraction (LVEF), left ventricle end diastolic volume (LVEDV), left ventricular end systolic volume (LVESV), and left ventricle mass (LVM). All of those measures didn't appear to be abnormal in average, as expected in a relatively healthy population such as the UK-Biobank participants. All baseline characteristics are summarized in Table 1.

**Table 1:** Clinical characteristics of LVH patients.

	Increased LVM (*N* = 4,255)
Age	64.2 ± 7.4
Female	(57.5)
BMI, kg/m^2^	28.2 ± 4.96
BSA, m^2^	1.9 ± 0.218
Diabetes mellitus	(8.3)
Hypertension	(47.3)
Dyslipidemia	(30.3)
Prior MI	(3.9)
CHF	(1.4)
PVD	(26)
Chronic lung disease	(2.8)
CKD	(0.9)
Aortic stenosis	(0.8)
Mitral regurgitation	(0.6)
Atrial fibrillation	(3)
LVEF, %	55.4 ± 7.3
LVEDV, ml	148 [125–175]
LVESV, ml	64 [52–80]
LVM index, g/m^2^	70 [60–78]
AST, U/L	24.4 [21–29]
ALT, U/L	20.5 [15.5–27.7]
Urea, mmol/L	5.28 [4.53–6.12]
Creatinine, µmol/L	69 [60.6–78.7]
HbA1c, mmol/L	34.9 [32.5–37.4]
HDL, mmol/L	1.40 [1.17–1.69]
LDL, mmol/L	3.52 [2.99–4.11]
Urate, µmol/L	302 [251–360]

Values are mean ± SD, (%), or median [Interquartile range]. ALT, alanine transaminase; AST, aspartate transaminase; BMI, body mass index; BSA, body surface area; CHF, congestive heart failure; CKD, chronic kidney disease; HbA1c, glycated haemoglobin; HDL, high density lipoprotein; LDL, low density lipoprotein; LVEDV, left ventricular end diastolic volume; LVEF, left ventricular ejection fraction; LVESV, left ventricular end systolic volume; LVM, left ventricle mass; MI, myocardial infraction; PVD, peripheral vascular disease.

### Unsupervised clustering results

**Figure 1:**
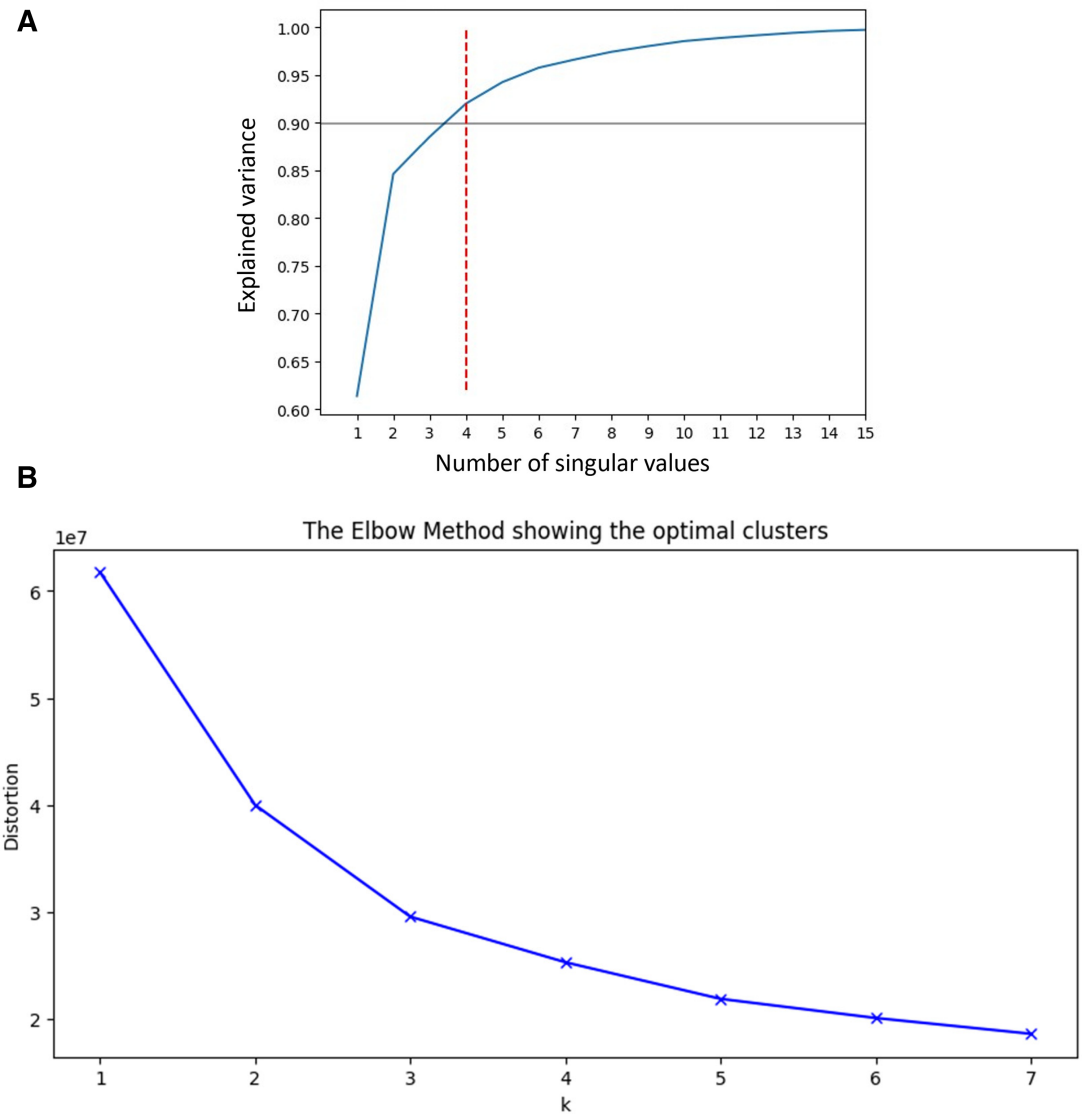
(**A**) SVD and explained variance. This plot shows the number of singular values needed to explain the variance of data across our cohort, suggesting that 4 values will be sufficient to explain more than 90% of variablity. SVD, Singular Value Decomposition. (**B**) Elbow plot. Elbow plot is a graphical method for finding the optimal value of clusters in clustering algorithms. This demonstrates that the number of clusters our cohort can be divided to is between 3 and 4, as there is only little added value of lowering the distortion beyond these number of clusters.

**Figure 2:**
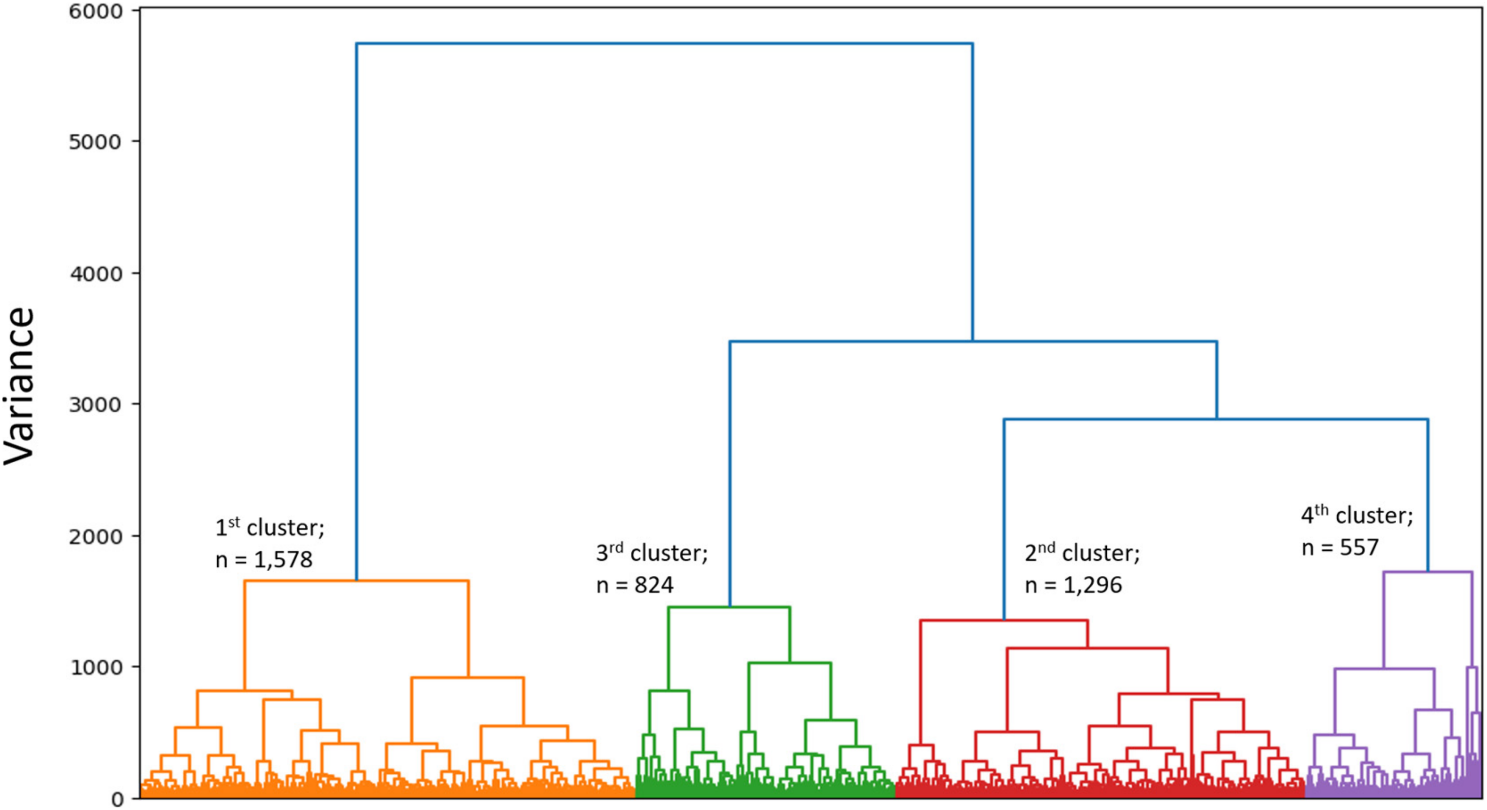
Dendrogram. Dendrogram is showing the hierarchial relationship between clusters and the optimal clustering of individuals who are relatively similar to each other.

SVD based methods suggested that a total of three or four clusters would be appropriate for this study population. We then employed different algorithms including the Silhouette Coefficient, the Calinski-Harabasz index, and the Davies-Bouldin index. Those algorithms, together with expert opinion, had directed us to make the final choice of 4 clusters (see Figures 1A,B and Table 2). The agglomerative clustering identified 4 distinct clusters, as depicted in the dendrogram (Figure 2). The 1st and the 2nd clusters were larger compared to the later two. Comparisons of baseline characteristics across clusters are provided in Table 3.

**Table 2:** Measures for quality of clustering.

# of clusters	Silhouette coefficient	Calinski Harabasz	Davies Bouldin
C = 3	0.235	**1,983** **.** **25**	1.37
C = 4	**0**.**244**	1,893.32	**1**.**25**

Bold values represent the better measure for quality of clustering. In Silhouette coefficient and Clinski Harabasz it is the bigger value, whereas in Davies Bouldin it is the smaller one.

**Table 3:** Clinical characteristics across clusters.

	Cluster 1	Cluster 2	Cluster 3	Cluster 4
	(*N* = 1,578)	(*N* = 1,296)	(*N* = 824)	(*N* = 557)
Age	64.2 ± 7.29	65.0 ± 7.2	63.8 ± 7.53	63.3 ± 7.84
Female	(93.1)	(62.7)	(10.7)	(14)
BMI, kg/m^2^	26.3 ± 4.33	29.6 ± 5.25	30.1 ± 4.8	27.7 ± 4.06
BSA, m^2^	1.77 ± 0.168	1.91 ± 0.194	2.08 ± 0.185	2.03 ± 0.176
Diabetes mellitus	(6.1)	(10.5)	(10.3)	(6.5)
Hypertension	(39.4)	(52.6)	(54.5)	(46.5)
Dyslipidemia	(21.6)	(33.3)	(41)	(32.5)
Prior MI	(1.5)	(3.5)	(6.3)	(8.3)
CHF	(0.5)	(1.4)	(1.9)	(3.4)
PVD	(23.7)	(27.9)	(28.9)	(23.7)
Chronic Lung disease	(1.8)	(4.1)	(2.9)	(2.5)
CKD	(0.3)	(0.9)	(1.8)	(1.3)
Aortic Stenosis	(0.4)	(1.2)	(0.7)	(0.9)
MitralRegurgitation	(0.4)	(0.7)	(0.7)	(1.1)
Atrial Fibrillation	(2.3)	(2.1)	(4.0)	(5.9)
LVEF, %	56.6 ± 6.18	56.7 ± 6.32	54 ± 7.38	50.7 ± 9.58
LVEDV, ml	133 [116–150]	137 [120–158]	169 [151–192]	209 [193–231]
LVESV, ml	56 [48–66]	59 [50–70.3]	77 [65.8–91]	99 [86–117]
LVM index, g/m^2^	60.1 [57–65]	67.4 [59–75.9]	77.5 [74–83.2]	78.8 [74.2–85.7]
AST, U/L	22.8 [19.8–26.2]	24.3 [21.1–29.2]	27.5 [23.8–33.1]	25.5 [22.2–29.3]
ALT, U/L	16.9 [13.6–21.9]	22 [16.4–28.9]	26.8 [20.1–36]	21.1 [16.6–27.8]
Urea, mmol/L	4.94 [4.2–5.74]	5.37 [4.68–6.25]	5.7 [4.9–6.54]	5.36 [4.66–6.14]
Creatinine, µmol/L	61.8 [55.9–68]	69.2 [61.3–78.2]	81.2 [73.5–89.1]	76.5 [68.8–82.5]
HbA1c, mmol/L	34.6 [32.2–37.1]	35.3 [32.8–38]	35.3 [32.9–37.9]	34.3 [32.3–36.8]
HDL, mmol/L	1.6 [1.34–1.86]	1.35 [1.14–1.59]	1.23 [1.04–1.44]	1.33 [1.13–1.61]
LDL, mmol/L	3.48 [2.99–4.03]	3.57 [3.01–4.2]	3.62 [3.04–4.16]	3.39 [2.87–3.93]
Urate, µmol/L	236 [208–260]	323 [303–348]	412 [388–452]	310 [276–337]

Values are mean ± SD, (%), or median [Interquartile range]. All variables had *p* < 0.05. ALT, alanine transaminase; AST, aspartate transaminase; BMI, body mass index; BSA, body surface area; CHF, congestive heart failure; CKD, chronic kidney disease; HbA1c, glycated haemoglobin; HDL, high density lipoprotein; LDL, low density lipoprotein; LVEDV, left ventricular end diastolic volume; LVEF, left ventricular ejection fraction; LVESV, left ventricular end systolic volume; MI, myocardial infraction; PVD, peripheral vascular disease.

### Clusters exploration

Cluster 1 consisted on 1,578 patients, almost all were females (93%). The average age was similar to the entire population, and the BMI was lower—with an average of 26 (4). Patients in this cluster had the lowest prevalence of co-morbidities, including co-morbidities that are known to be cardiovascular risk factors such as diabetes mellitus (6%), hypertension (39%), dyslipidemia (22%), and chronic kidney disease (0.3%). The median of all cardiac measures was the lowest, in comparison to all other clusters, and none of the laboratory tests was abnormal. Therefore, this cluster served as the baseline for all of our comparisons.

Cluster 2 (*n* = 1,296) also included mainly females (63%), with a slightly higher age than the other clusters [average 65(7)]. Patients in this cluster had more co-morbidities compared to cluster 1, with the highest prevalence of diabetes mellitus (10.5%), and chronic lung disease (4%). In similar to the first cluster, cardiac measures were low, pointing the lack of overt structural heart disease among those patients. None of the laboratory tests was abnormal.

Cluster 3 (*n* = 824) was the cluster with the most co-morbidities and cardiovascular risk factors, including highest portion of males (89%), obesity [average BMI 30 (5)], diabetes mellitus (10.3%), hypertension (54.5%), dyslipidemia (41%), peripheral vascular disease (29%), and chronic kidney disease (1.8%). Not surprisingly, this was also the cluster with the most abnormal laboratory tests including liver function tests (AST, ALT), kidney function tests (Urea, Creatinine), Urate, and lipid profile (lowest HDL and the highest LDL). In terms of cardiac measures, this cluster showed lower values than the first two, but much better measures compared to the 4th cluster.

Cluster 4 (*n* = 557) was the most interesting one. It was also consisted mainly of males (86%), with an average age of 63 (8). This cluster had higher prevalence of co-morbidities comparing to the 1st cluster, but less than the 2nd and 3rd clusters. The cardiac measures of this cluster were the most abnormal—indicating pathological changes in the structure of the heart which are expressed by the left ventricular volumes [LVEDV median 209 (193–231), LVESV median 99 (86–117)], the left ventricle mass [LVM index median 79 (74–86)], and function [LVEF 51(10)]. In conjunction with those findings, this cluster exhibited the highest prevalence of prior myocardial infraction (8.3%), and prior diagnosis of congestive heart failure (3.4%).

**Figure 3:**
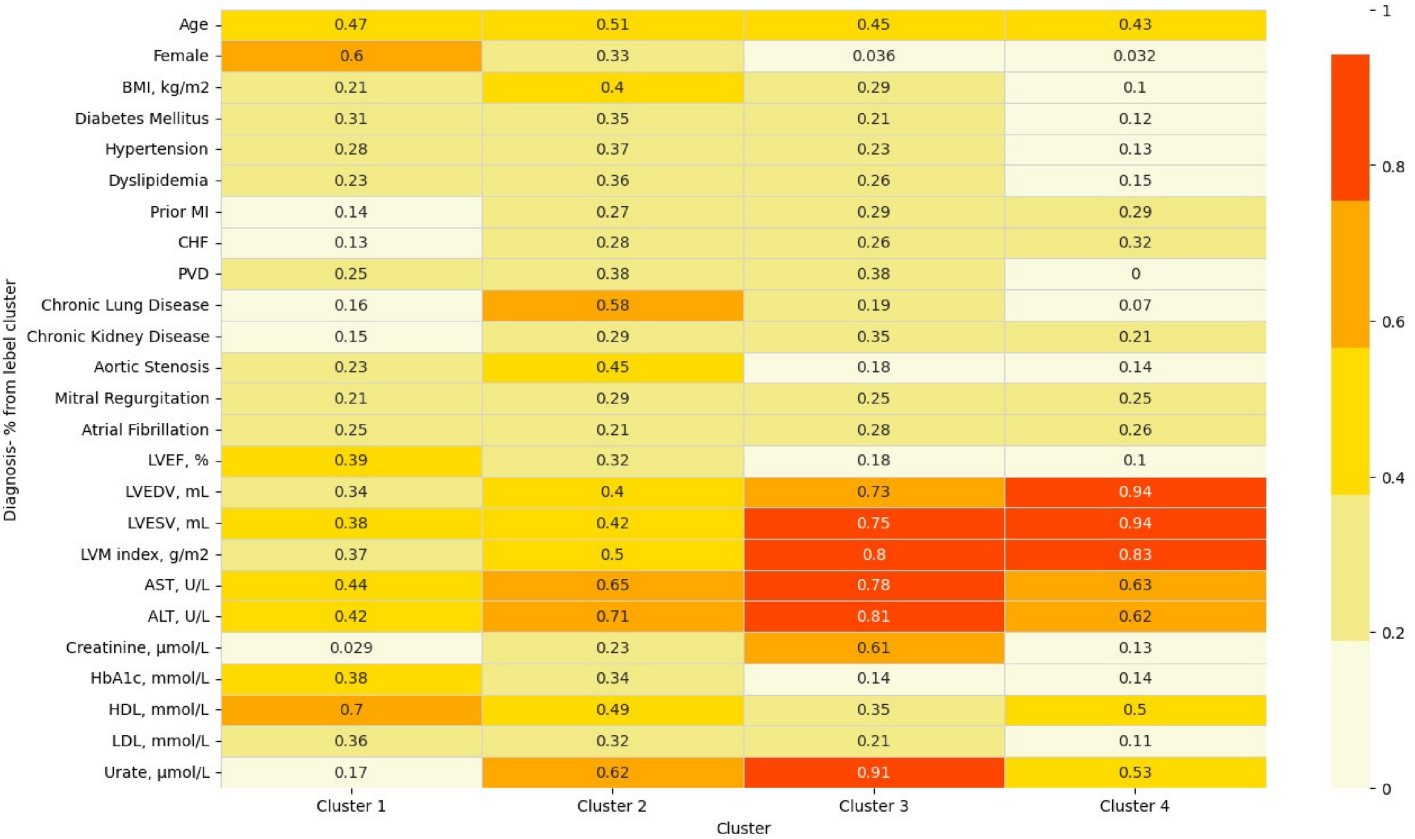
Heatmap of variables across clusters. This heatmap demonstrates the uniqueness of each cluster by highlighting the relative ratio of each binary variable/quantile of mean for numeric features. ALT, alanine transaminase; AST, aspartate transaminase; BMI, body mass index; CHF, congestive heart failure; HbA1c, glycated haemoglobin; HDL, high density lipoprotein; LDL, low density lipoprotein; LVEDV, left ventricular end diastolic volume; LVEF, left ventricular ejection fraction; LVESV, left ventricular end systolic volume; MI, myocardial infraction; PVD, peripheral vascular disease.

A variable heatmap that underscores the differences between clusters is presented in Figure 3.

### The association between clusters and outcomes during follow-up

During a median follow-up period of 5 years (IQR: 4-6), 100 patients (2%) died, 118 (2.8%) were admissioned due to heart failure, 29 (0.7%) were admissioned due to ventricular arrhythmia, and 208 (5%) were admissioned due to atrial fibrillation.

**Figure 4:**
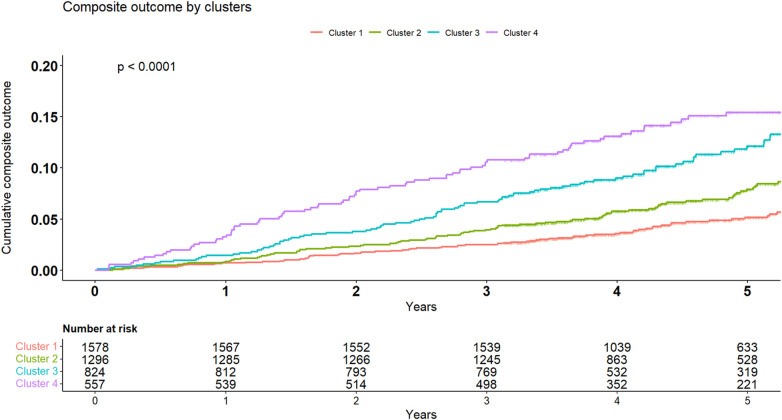
Kaplan-Meier for composite endpoint. Kaplan-Meier cumulative incidence curves of the composite outcome demonstrating a graded incidence of cardiovascular outcomes across clusters. Log rank *p* < 0.001.

The crude number of events in each cluster is specified in Tables 4A,B. Kaplan-Meier survival analysis revealed that the cumulative probability of the composite endpoint at 5 years of follow-up was 5.2%±0.6%, 8%±0.9%, 1.2%±1.3%, and 15.5%±1.7% for each cluster, respectively (Figure 4; *p* Log rank <.001 for the overall difference during follow-up). Univariate Cox analysis revealed that compared to the 1st cluster, patients in the 2nd, 3rd, and 4th clusters had 60%, 104%, and 164% increased risk of a major event, respectively (95% CI 1.2–2.16, *p* < .001 for cluster 2; 95% CI 1.49–2.78, *p* < .001 for cluster 3; 95% CI 1.92–3.62, *p* < .001 for cluster 4). Multivariate Cox analysis adjusted for LVM index, which is the most contributing factor for mortality among increased LVM patients, consistently demonstrated that compared to the 1st cluster, patients in the 2nd, 3rd, and 4th clusters had 38%, 49%, and 94% increased risk of a major event, respectively (95% CI 1.02–1.88, *p* = 0.039 for cluster 2; 95% CI 1.06–2.10, *p* = 0.022 for cluster 3; 95% CI 1.36–2.78, *p* < .001 for cluster 4). Every 10 g/m^2^ increase in LVM index was associated with 22% increased risk of a major event (95% CI 1.12–1.33, *p* < .001).

**Table 4A:** Univariate Cox analysis for composite endpoint.

	HR	95% CI	*p* value
Unsupervised clusters^a^ [*n* events (%)]:			
Cluster 1 [*n* = 80 (5%)]	Reference		
Cluster 2 [*n* = 99 (7.6%)]	1.6	1.2–2.16	**<0**.**001**
Cluster 3 [*n* = 97 (11.7%)]	2.04	1.49–2.78	**<0**.**001**
Cluster 4 [*n* = 88 (15.8%)]	2.64	1.92–3.62	**<0**.**001**

Bold values represent statistical significance (*p* < 0.05).

^a^ Patients in cluster 1 are serving as baseline.

**Table 4B:** Multivariate Cox analysis for composite endpoint.

	HR	95% CI	*p* value
Unsupervised clusters^a^ [*n* events (%)]:			
Cluster 1 [*n* = 80 (5%)]	reference		
Cluster 2 [*n* = 99 (7.6%)]	1.38	1.02–1.88	**0**.**039**
Cluster 3 [*n* = 97 (11.7%)]	1.49	1.06–2.1	**0**.**022**
Cluster 4 [*n* = 88 (15.8%)]	1.94	1.36–2.78	**<0**.**001**
LVM indexed, per 10 g/m^2^	1.22	1.12–1.33	**<0**.**001**

Bold values represent statistical significance (*p* < 0.05).

^a^ Patients in cluster 1 are serving as baseline; LVM, left ventricle mass.

**Figure 5:**
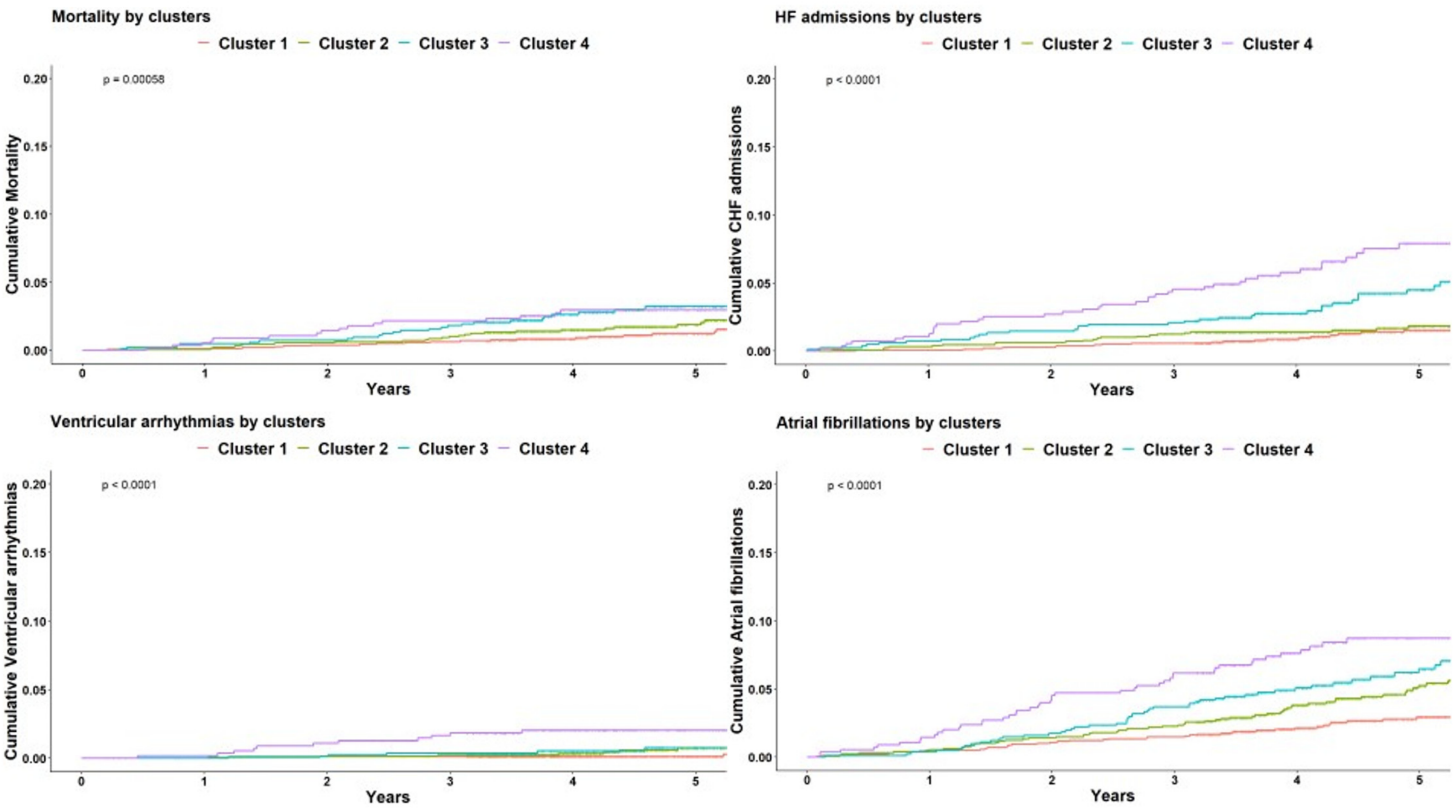
Kaplan-Meier for each outcome. Kaplan-Meier cumulative incidence curves for each of the cardiovascular outcome comprising the composite endpoint, demonstrating a graded incidence across clusters. Log rank *p* < 0.001 for all. HF, heart failure.

Breaking down the composite outcome to each endpoint, the most common outcomes were admissions due to heart failure [*n* = 118 (2.8%)] and atrial fibrillation [*n* = 208 (5%)]. The association of all clusters with each endpoint was consistent with the composite endpoint as seen in Figure 5. However, because of the relatively small rate of events, certain clusters were underpowered, especially cluster 2 which did not demonstrated statistical significance with any endpoint. A summary of crude number of events for each outcome, as well as their association with all clusters are presented in Table 5.

**Table 5:** Univariate Cox analysis for each endpoint.

Unsupervised clusters [*n* events (%)]:	HR	95% CI	*p* value
All-cause mortality			
Cluster 1 [*n* = 22 (1.4%)]	reference		
Cluster 2 [*n* = 27 (2%)]	1.50	0.85–2.63	0.2
Cluster 3 [*n* = 30 (3.6%)]	2.64	1.53–4.59	**<0**.**001**
Cluster 4 [*n* = 21 (3.7%)]	2.70	1.48–4.91	**<0**.**001**
HF admissions			
Cluster 1 [*n* = 22 (1.4%)]	reference		** **
Cluster 2 [*n* = 24 (1.9%)]	1.32	0.74–2.36	0.3
Cluster 3 [*n* = 32 (3.9%)]	2.84	1.65–4.89	**<0**.**001**
Cluster 4 [*n* = 40 (7.2%)]	5.27	3.13–8.86	**<0**.**001**
Ventricular arrhythmia			
Cluster 1 [*n* = 3 (0.2%)]	reference		** **
Cluster 2 [*n* = 8 (0.6%)]	3.23	0.86–12.2	0.083
Cluster 3 [*n* = 6 (0.7%)]	3.85	0.96–15.4	0.057
Cluster 4 [*n* = 12 (2.1%)]	11.3	3.20–40.2	**<0**.**001**
Atrial fibrillation			
Cluster 1 [*n* = 46 (3%)]	reference		** **
Cluster 2 [*n* = 62 (4.8%)]	1.65	1.12–2.41	**0**.**01**
Cluster 3 [*n* = 51 (6.2%)]	2.17	1.46–3.23	**<0**.**001**
Cluster 4 [*n* = 49 (8.8%)]	3.10	2.07–4.63	**<0**.**001**

Bold values represent statistical significance (*p* < 0.05).

**Figure 6:**
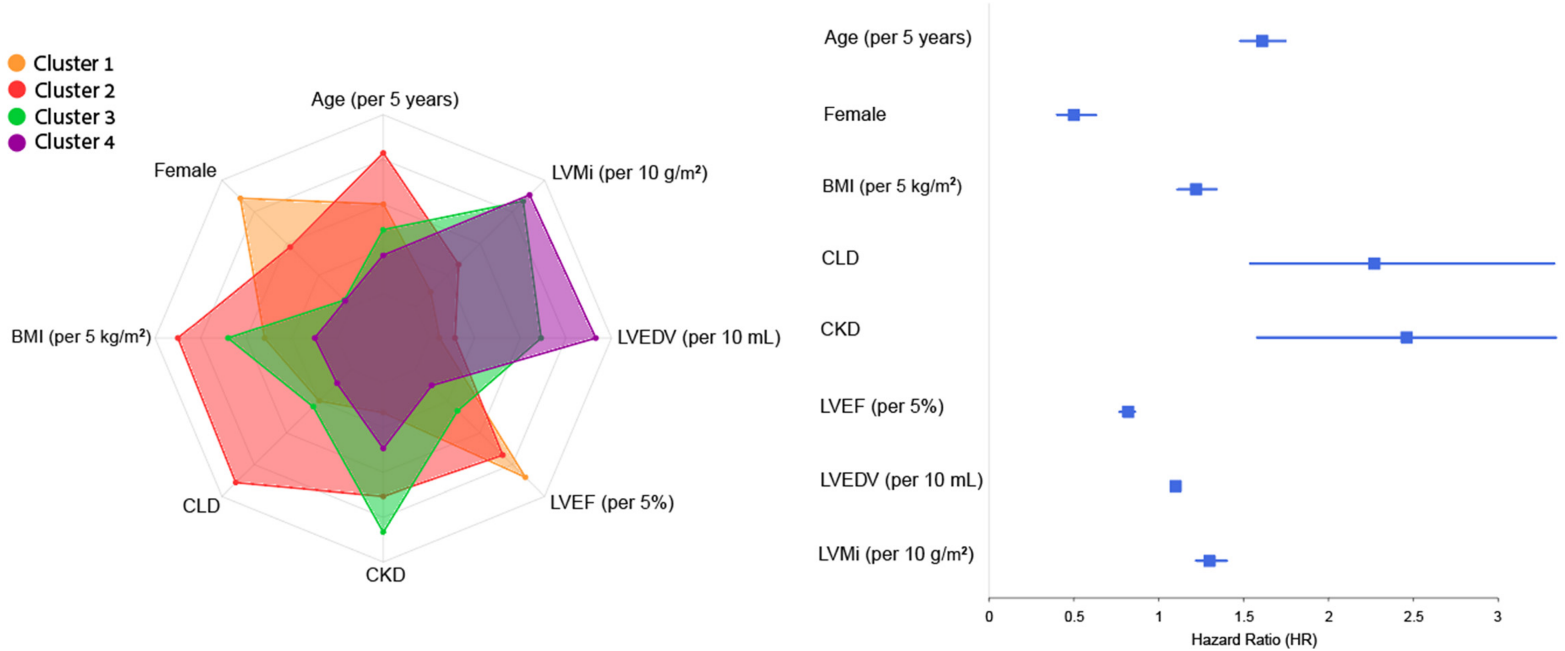
Radar and forest plots of variables contribution to composite outcome across clusters. This plot depicts the eight features that differ the most between clusters, and their contribution to risk of the composite outcome (as showed in the forest plot by calculating the HR and 95% CI of each feature in a univariate Cox analysis), thus linking between clusters to cardiovascular risk and providing further explainability to the association of the four clusters with clinical outcomes. BMI, body mass index, CKD, chronic kidney disease, CLD, chronic lung disease; LVEDV, left ventricular end diastolic volume; LVEF, left ventricular ejection fraction; LVMi, left ventricular mass index.

Lastly, in order to analyze the relationship between clustering results and clinical outcomes we have identified the features that differ the most between clusters, and evaluated their association with the composite outcome. These features and corresponding risk for the composite outcome are as follows: age (HR 1.61, 95% CI 1.48–1.75, *p* < .001), female sex (HR 0.5, 95% CI 0.4–0.63, *p* < .001), BMI [per 5 kg/m^2^] (HR 1.22, 95% CI 1.11–1.34, *p* < .001), chronic lung disease (HR 2.27, 95% CI 1.54–3.33, *p* < .001), chronic kidney disease (HR 2.46, 95% CI 1.58–3.34, *p* < .001), LVEF [per 5%] (HR 0.82, 95% CI 0.77–0.86, *p* < .001), LVEDV [per 10 ml] (HR 1.1, 95% CI 1.08–1.12, *p* < .001), and LVM index [per 10 g/m^2^] (HR 1.3, 95% CI 1.22–1.39, *p* < .001). Figure 6 depicts the difference (prevalence for binary variables and quantile of mean for numeric variables) of these features across clusters, as demonstrated in the radar plot, together with a forest plot that summarizes the contribution of each feature for the risk of adverse cardiovascular outcome.

## Discussion

In this study, we used cluster analysis to identify the differences between populations with increased LVM, revealing four groups with distinct clinical, laboratory and cardiac features. The clusters were associated with different clinical outcome, defined by the composition of all-cause mortality, HF admissions, ventricular arrhythmia and new-onset AF. Using the clinical clustering, as opposed to the echocardiographic clustering as done in the DHS, may help improve the management of these patients, as mild changes in the LVM may represent their clinical course.

We observed that cluster 1 patients correspond to the “intermediate hypertrophy” group in the DHS. It consisted mainly of women with almost normal LVM and a low prevalence of IHD. This group is still an enigma, as the reason for increased LVM is obscure, and their prognosis is favorable. Previously, high prevalence of increased LVM in women was not found to be associated with age, hypertension, or obesity (24), suggesting another pathophysiology to this idiopathic observation. Sex-hormones were previously suggested as mediators of LVM (25), potentially segregating men and women without other risk factors.

Cluster 2 was also female-dominant, with higher LVM, higher BMI, and prevalence of lung disease, HTN, DM, AS, and CHF. Previous studies found association between these co-morbidities in addition to low prevalence of IHD, and HfpEF (26, 27). Hence, this cluster may represent patients who either have or will develop HFpEF.

Cluster 3 is male-predominant, with the highest BMI and worse metabolic syndrome features, including HTN and the expected renal damage, with a mild reduction in LVEF and high LVM. This cluster corresponds to and shares features with “thick” or “thick and dilated” groups in the DHS. HTN, metabolic syndrome and CKD are all associated with concentric hypertrophy and poor outcomes (28, 29), and this cluster may represent the early phase of these patients.

Cluster 4 is also male-predominant and classically represents the “dilated” group in the DHS with the highest LVM, LVEDV, and LVESV, and the highest prevalence of IHD, MR, and AF These conditions were widely described as factors contributing to eccentric hypertrophy (30–32). This cluster represents the population in the early phases of the vicious cycles between their LV and their predisposing conditions (33). As concentric remodeling is the main hemodynamic response to HTN, eccentric changes are associated with IHD in patients with HTN, representing the beginning of the systolic failure (34). This cluster has the poorest outcomes as it combines the highest prevalence of serious conditions.

The distinction between the groups regarding clinical outcomes is significant, although the study population was relatively healthy, with mild elevation in the LVM. Previous studies could not associate clinical parameters and hard endpoints, emphasizing the need for new approaches.

Machine learning (ML)-based analysis is gaining momentum with various uses. In HF, a supervised ML model that included leg bioimpedance, age, sex, and self-reported myocardial infarction accurately predicted HF without predefining the variables (35). Cluster analysis used in the HFpEF population was able to distinguish between three phenogroups, with clinical and prognostic differences (18). In patients with HF with recovered EF, unsupervised cluster analysis helped distinguish between groups with different characteristics and outcomes, providing information regarding those who are at higher risk of HF recurrence and all-cause mortality (17). ML algorithm utilizing both advanced echocardiographic data of full heart cycle and clinical parameters was able to predict response to CRT implantation in HF patients (16). Cluster analysis applied to a cohort of patients with at least moderate TR identified phenotypes with different mortality risks, suggesting underlying pathophysiology (36).

These examples, along with our findings, shed some light on the future potential risk stratification of different diseases. Non-classic risk factors, which sometimes are not intuitive when conducting a study, may be found by unsupervised ML algorithms, improving the management of patients. Large databases already in use may serve as potential grounds for novel approaches in diagnosing and treating various conditions. These advances should be used to tailor a specific and more efficient treatment for patients based on their classical attributes and overlooked ones.

### Study strengths and limitations

A major strength of this study lies in its participants, encompassing a large and well-documented population from the UK Biobank. Additionally, the exploratory analysis of clusters characteristics and their link to adverse events, validates the demonstrated association between the unsupervised clusters and observed outcomes, and may suggest that the ML algorithm did not function as a black box for this clustering task. This study has several important limitations. First, our unsupervised clustering model was based on data exclusively from the UK Biobank, and currently lacks external validation, which limits the generalizability of our findings and introduce a possible selection bias. Second, important clinical and laboratory predictors of poor survival such as clinical signs of symptoms heart failure, NYHA functional class, BNP, troponin, and more laboratory markers were not available, potentially limiting the comprehensiveness of the cluster analysis. Lastly, while independent associations between clusters and clinical outcomes have been demonstrated, causality could not be established due to study design.

## Conclusions

Utilizing unsupervised cluster analysis on a large patient cohort exhibiting elevated left ventricular mass (LVM), we discerned four distinct phenotypes characterized by unique clinical and imaging attributes, each yielding divergent outcomes. Machine learning algorithms offer a promising avenue for novel insights into the associations between predisposing conditions and diseases, providing advanced and unparalleled perspectives.

## Data Availability

Publicly available datasets were analyzed in this study. This data can be found here: UK Biobank (application 96272).
